# Infective Diseases

**Published:** 1896-01-11

**Authors:** 


### PROGRESS IN MEDICINE.

### INFECTIVE DISEASES.

**Scarlatina.**—Secondary rashes may be due to several causes. Thus convalescents are apt to exhibit a rash like the primary one when anything causes a rise of temperature, such as tonsillitis or acute nephritis, but these attacks are not followed by desquamation and other symptoms. In a few cases the rash is a symptom of a genuine relapse. A. Griffith,[1] of the Bagthorpe Fever Hospital, found fourteen such instances among 2,000 cases of scarlatina, and noticed the special liability to nephritis, which occurs after these second attacks. It is curious, too, that when the interval between the attacks was very brief, the first attack had usually been of mild character; and he raises the question whether the relapse is a self-infection, or whether it is caused by the presence of other patients suffering from a fever of a more virulent type. On the latter supposition true relapses should not occur where only single patients are treated. With regard to the infectiousness of convalescent patients, and the occasional reappearance of the disease in a family after their return from a hospital, Caiger[2] points out that after the peeling is complete, any discharge from nose or ears is infectious, and that probably the breath of a healthy person, after prolonged residence in a fever ward, is capable of conveying the disease. Even the eucalyptus treatment does not seem entirely to prevent these occasional "return" cases, though, according to Priestley,[3] who gave it an exhaustive trial with 120 patients, its use is followed by a lower death-rate, fewer complications, and a shorter average time in the hospital. Suppurative joint lesions are very occasionally found in scarlatinal rheumatism, but in some epidemics they may be less rare. Thus, while Hodges met with none in 7,000 cases during three years, Mary Sturge and C. B. Smith[4] record nine in 5,000 attacks at the North-Eastern Fever Hospital. These joint troubles seem to appear most often about the third week. When early, they are pyæmic in origin; when later, they may develop from the ordinary serous arthritis. Some focus of suppuration in the fauces or elsewhere is often found to be their cause.

In various obscure cases of fever, where none of the ordinary fever-producing diseases can be discovered, W. Carter[5] has drawn attention to the importance of searching for any small ulcer which may be present and healing it, since frequently from a small septic area of this kind toxines may be absorbed sufficient to produce the symptoms of fever. He describes cases where long-continued and inexplicable fever, recurring in periods of about a week, followed by an intermission of four or five days, was traced to the presence of a piece of necrosed bone in the posterior nares, and disappeared when this was removed.

**Actinomycosis.**—Rydygier[6] discusses the value of local injections of potassium iodide, which he thinks will often thus effect a cure where it fails if given by the mouth, and in cases where complete excision is impossible. Van Arsdale[7] records a case where the club form of the germ was absent, and only threads similar to leptothrix could be seen.

**Cerebro Spinal Meningitis.**—Various observers[8] have noticed the presence of Fränkel's pneumococcus in the blood, urine, and fæces during attacks, even those of a mild type. It had been previously supposed, on the other hand, that the microbe was only present in severe cases. Bight[9] has employed serum from a convalescent case as an injection with apparent success. A single dose of 5 c. cm. was administered to a child of seven years in whom the diptococcus had been demonstrated.

**Beri Beri.**—Besides the outbreaks at Dublin and in an English asylum, Baker[10] describes a recent epidemic in the lunatic asylum at Rangoon, where 211 people were attacked, with at least seven deaths. The danger of outbreaks occurring in overcrowded and insanitary communities appears to be great when the infection exists in the country. From this point of view its occurrence in widely separated localities in Australia is ominous. Corlette[11] has just communicated an account of 60 cases at Sydney among Chinese and Orientals, many of whom had resided there for years. Besides the well-known peripheral neuritis, œdema, and dyspnœa, he remarks the frequent attacks of sweating, and the existence of two acutely tender spots on the foot, one on the dorsum and one on the outer side. There is reason to fear that the disease will ere long appear among the European races of that country.

[1] N.Y. Med. J., Nov. 9. [2] Lancet, Oct. 12. [3] Thur. Gaz., Aug. 15. [4] Lancet, Nov. 16. [5] Lancet, Oct. 19. [6] B. M. J., Oct. 19. [7] Annals of Surg., Sept. [8] B. M. J., Oct. 5. [9] B. M. J., Nov. 9. [10] B. M. J., Sept. 28

